# Deciphering STAT3 signaling potential in hepatocellular carcinoma: tumorigenesis, treatment resistance, and pharmacological significance

**DOI:** 10.1186/s11658-023-00438-9

**Published:** 2023-04-21

**Authors:** Mehrdad Hashemi, Eisa Sabouni, Parham Rahmanian, Maliheh Entezari, Mahsa Mojtabavi, Behnaz Raei, Mohammad Arad Zandieh, Mitra Behroozaghdam, Sepideh Mirzaei, Kiavash Hushmandi, Noushin Nabavi, Shokooh Salimimoghadam, Jun Ren, Mohsen Rashidi, Rasoul Raesi, Afshin Taheriazam, Athanasios Alexiou, Marios Papadakis, Shing Cheng Tan

**Affiliations:** 1 Farhikhtegan Medical Convergence Sciences Research Center, Farhikhtegan Hospital Tehran Medical Sciences, Islamic Azad University, Tehran, Iran; 2 Department of Genetics, Faculty of Advanced Science and Technology, Tehran Medical Sciences, Islamic Azad University, Tehran, Iran; 3 Faculty of Veterinary Medicine, Science and Research Branch, Islamic Azad University, Tehran, Iran; 4 Mashhad Branch, Islamic Azad University, Mashhad, Iran; 5 Division of Epidemiology, Department of Food Hygiene and Quality Control, Faculty of Veterinary Medicine, University of Tehran, Tehran, Iran; 6 Department of Biology, Faculty of Science, Science and Research Branch, Islamic Azad University, Tehran, Iran; 7 Department of Urologic Sciences and Vancouver Prostate Centre, University of British Columbia, Vancouver, BC V6H3Z6 Canada; 8 Department of Biochemistry and Molecular Biology, Faculty of Veterinary Medicine, Shahid Chamran University of Ahvaz, Ahvaz, Iran; 9 Department of Cardiology, Zhongshan Hospital, Shanghai Institute of Cardiovascular Diseases, Fudan University, Shanghai, 200032 China; 10 Department Pharmacology, Faculty of Medicine, Mazandaran University of Medical Sciences, Sari, Iran; 11 The Health of Plant and Livestock Products Research Center, Mazandaran University of Medical Sciences, Sari, Iran; 12 Department of Health Services Management, Mashhad University of Medical Sciences, Mashhad, Iran; 13 Department of Medical-Surgical Nursing, Mashhad University of Medical Sciences, Mashhad, Iran; 14 Department of Orthopedics, Faculty of Medicine, Tehran Medical Sciences, Islamic Azad University, Tehran, Iran; 15 Department of Science and Engineering, Novel Global Community Educational Foundation, Hebersham, Australia; 16 AFNP Med Austria, Vienna, Austria; 17 Department of Surgery II, University Hospital Witten-Herdecke, University of Witten-Herdecke, Heusnerstrasse 40, 42283 Wuppertal, Germany; 18 UKM Medical Molecular Biology Institute, Universiti Kebangsaan Malaysia, Kuala Lumpur, Malaysia

**Keywords:** Hepatocellular carcinoma, Liver cancer, Noncoding transcripts, STAT3, Molecular signaling

## Abstract

Hepatocellular carcinoma (HCC) is considered one of the greatest challenges to human life and is the most common form of liver cancer. Treatment of HCC depends on chemotherapy, radiotherapy, surgery, and immunotherapy, all of which have their own drawbacks, and patients may develop resistance to these therapies due to the aggressive behavior of HCC cells. New and effective therapies for HCC can be developed by targeting molecular signaling pathways. The expression of signal transducer and activator of transcription 3 (STAT3) in human cancer cells changes, and during cancer progression, the expression tends to increase. After induction of STAT3 signaling by growth factors and cytokines, STAT3 is phosphorylated and translocated to the nucleus to regulate cancer progression. The concept of the current review revolves around the expression and phosphorylation status of STAT3 in HCC, and studies show that the expression of STAT3 is high during the progression of HCC. This review addresses the function of STAT3 as an oncogenic factor in HCC, as STAT3 is able to prevent apoptosis and thus promote the progression of HCC. Moreover, STAT3 regulates both survival- and death-inducing autophagy in HCC and promotes cancer metastasis by inducing the epithelial–mesenchymal transition (EMT). In addition, upregulation of STAT3 is associated with the occurrence of chemoresistance and radioresistance in HCC. Specifically, non-protein-coding transcripts regulate STAT3 signaling in HCC, and their inhibition by antitumor agents may affect tumor progression. In this review, all these topics are discussed in detail to provide further insight into the role of STAT3 in tumorigenesis, treatment resistance, and pharmacological regulation of HCC.

**Graphical Abstract:**

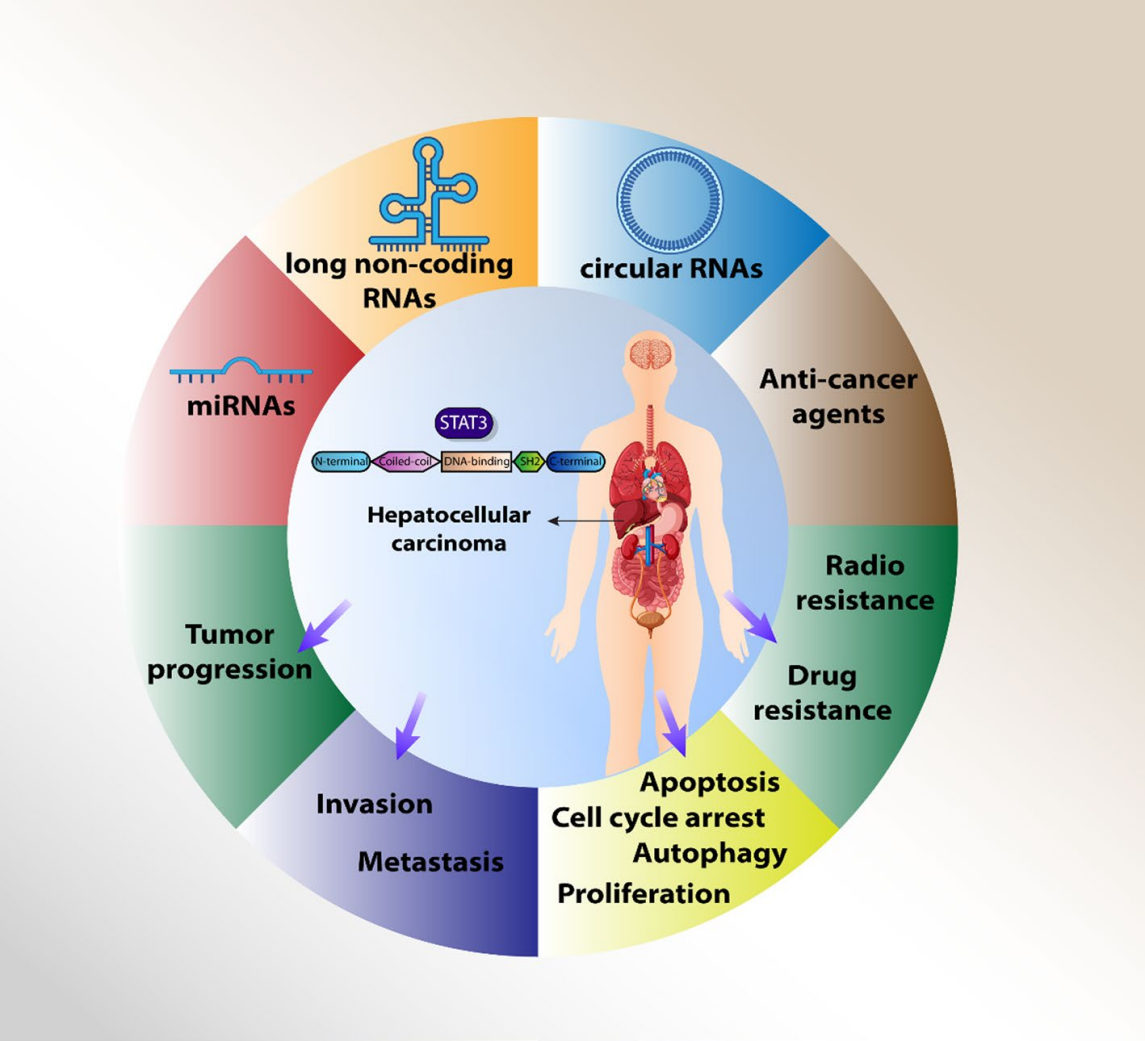

## Introduction

Liver cancer is the fifth most common tumor worldwide and the second leading cause of death [1]. In 2012 alone, a total of 14.1 million cases of liver cancer were diagnosed, which were responsible for 745,500 deaths [2]. The mortality rate of liver cancer differs between men and women. It is the second leading cause of death in men and the sixth leading cause of death in women. The most common form of liver cancer is hepatocellular carcinoma (HCC), which can be caused by hepatitis B virus (HBV) and hepatitis C virus (HCV) infection, aflatoxin contamination in food, alcohol consumption, obesity, type 2 diabetes, liver cirrhosis, and smoking, among other factors [2, 3]. Due to the synergy between these additional factors, the risk of developing HCC in the community and individuals has increased significantly [4–7]. Up to 70–85% of HCC cases are caused by HBV and HCV [8]. According to studies, 50% of HCC cases are due to HBV, while HCV is responsible for the development of 25% of HCC cases [8, 9]. Treatment strategies for HCC vary and include chemotherapy, radiotherapy, surgery, and immunotherapy. Clinical studies have shown that chemotherapy can improve the prognosis of patients with HCC. For example, a combination of atezolizumab and bevacizumab can increase the survival rate of patients with HCC, and its therapeutic potential is better than that of sorafenib [10]. In addition, a combination of oxaliplatin and fluorouracil shows a better effect in improving the prognosis of patients with HCC compared with sorafenib [11]. Basic research has shown that the function of chemotherapy in the treatment of HCC may be affected by the development of drug resistance [12, 13]. Moreover, there is a possibility that the response of HCC cells to radiotherapy may be altered. For example, high expression of METTL1 leads to DNA repair and prevents the radiosensitivity of HCC cells [14]. In addition, dysbiosis in the gut microbiota is responsible for the impairment of the antitumor immune response by radiotherapy and may enhance HCC progression [15]. Some genetic modulations may also increase the efficacy of immunotherapy in the treatment of HCC. For example, silencing of MCT4 increases T cell infiltration and promotes immunotherapeutic potential in suppressing HCC [16]. GDF1 is involved in the upregulation of CTA by downregulating LSD1 to improve the immunotherapy of HCC [17]. However, much progress still needs to be made in the treatment of patients with HCC. Therefore, one area for developing new therapeutics is to focus on factors that mediate the progression of HCC. The cellular and molecular interactions determine the progression of HCC cells via molecular signaling pathways [18–20]. Disruption of the intrahepatic microbiota leads to stimulation of hepatic stellate cells and their senescence, to direct liver cirrhosis toward HCC development [21]. Moreover, SNORAD17 inactivates p53 by binding to NPM1 and MYBBP1A in the nucleus to promote HCC progression [22]. Inhibition of IRF8 impairs HCC cell progression, which is important for increasing the potential of anti-PD-1 therapy [23]. The extracellular vesicles derived from hepatic stellate cells are able to secrete HK1 to increase the malignancy of HCC by stimulating glycolysis [24]. Even more interestingly, dysregulation of molecular signaling pathways may lead to drug resistance in HCC [25]. The upregulation of ROBO1 is considered to be a factor for the increase of HCC progression and its downregulation by miR-152-3p affects the malignancy of HCC [26]. In addition, inflammation is considered a factor in the pathogenesis of HCC, and the upregulation of signal transducer and activator of transcription 3 (STAT3) by GNAS creates such a condition [27]. Therapeutic targeting of UCK2 and its downregulation may lead to an increase in cancer immunity in HCC [28]. Since molecular interactions play a key role in HCC progression [29, 30], the current review was dedicated to understanding the function of STAT3 signaling in HCC tumorigenesis.

There are also a number of reviews on the STAT3 pathway in HCC [31–35]. However, their structure is not comprehensive, and the novelty of the current work is that it has focused in different sections and subsections on the role of STAT3 in growth, invasion, drug resistance, radioresistance, molecular pathways regulating STAT3, and its targeting by anticancer agents. These topics have not been fully investigated in previous reviews. As science continues to advance, an up-to-date review of STAT3 is needed for HCC. Therefore, most references in this article are new and updated.

## STAT3 signaling: an overview

### Structure and mechanism of activation

**Fig. 1:**
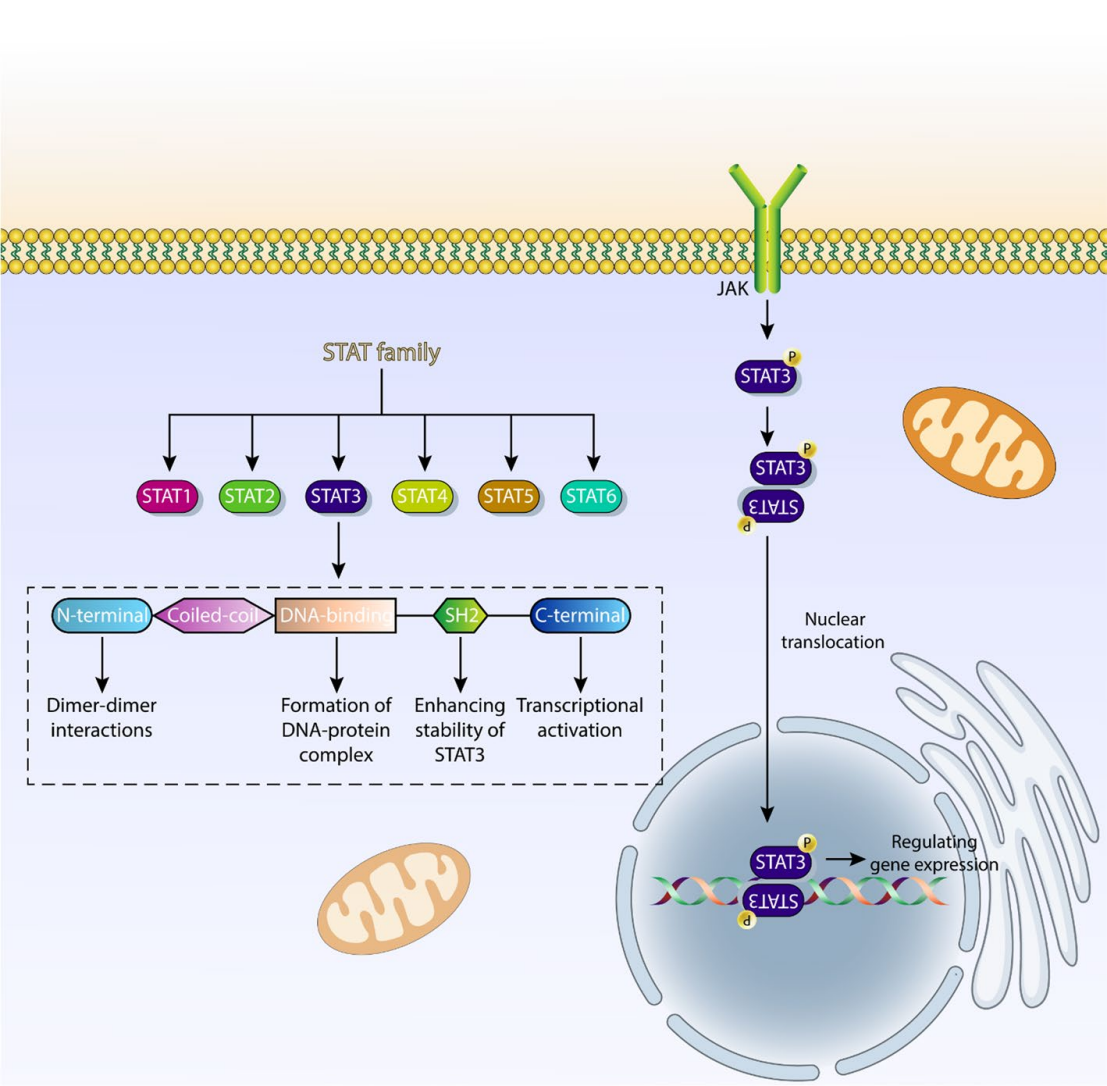
A schematic representation of STAT3 in cells

The STAT family comprises seven transcription factors: STAT1, STAT2, STAT3, STAT4, STAT5a, STAT5b, and STAT6. Their interaction with cytokines, growth proteins, and polypeptide ligands is critical for controlling important biological events in cells [36–38]. STAT3 is the best known member of the STAT family. This transcription factor has the ability to bind to DNA and its expression can be induced by cytokines, growth factors, inflammation, interleukin-6 (IL-6), and others [39]. Structurally, STAT3 has a unique shape and the presence of different domains in this protein leads to its specific functions in cells. The N-terminal, coiled-coil, DNA-binding, Src homology 2 (SH2), and C-terminal transactivation domains make up STAT3 [37, 38, 40]. Each of these domains is responsible for a specific function of STAT3. For example, dimer–dimer interactions are mediated by the N-terminal domain and the formation of the DNA–protein complex in STAT3 is mediated by the DNA-binding domain. The SH2 domain is involved in increasing the stability of STAT3 and transcriptional activation is mediated by the C-terminal domain [40]. STAT3 was first discovered in 1996, when researchers investigated the intracellular transduction of epidermal growth factor (EGF) and IL-6, and STAT3 was believed to be involved in the regulation of cell growth and inflammatory responses [41, 42]. As a downstream target of inflammatory factors and growth factors, STAT3 is able to regulate important biological mechanisms in cells such as proliferation, differentiation, migration, and others [43–46]. In cells, there are endogenous inhibitors of STAT3 signaling, including PIAS, SOCS, and protein tyrosine phosphatases, as well as ubiquitin enzymes that can suppress this pathway [47]. Stimulation of STAT3 signaling in cells is mediated by phosphorylation at tyrosine (705) and serine (727) residues induced by JAK proteins, tyrosine kinases, cytokines, and nonreceptor tyrosine kinases such as SRC and ABL. After phosphorylation of STAT3 and formation of homo- or heterodimers, STAT3 migrates to the nucleus to regulate gene expression [48]. Figure 1 illustrates STAT3 signaling in cells.

### STAT3 signaling in cancer

The field of oncology is rapidly evolving thanks to the development of various therapeutics. A major limitation of current treatment strategies is that there are few therapies based on targeting of molecular signaling pathways that regulate cancer progression. Therefore, due to the development of precision medicine and improvements in the biological field, it is strongly recommended to develop novel therapies based on molecular signaling pathways that are mainly involved in cancer development. There is increasing evidence that STAT3 regulation is important in cancer and promotes cancer progression. Exosomal S100A4 stimulates STAT3 signaling to mediate resistance of lung tumor cells to the immune system [49, 50]. Moreover, high expression of STAT3 mediated by IL-6 can promote invasion and metastasis of gastric cancer cells [51]. The presence of a high-fat diet due to cyclophilin B is significant in inducing STAT3 signaling to increase PVT1 expression. Moreover, there is a positive feedback loop between STAT3 and PVT1 that may promote the progression of colorectal tumor cells [52]. When STAT3 expression increases, it induces YAP signaling to promote lung tumor cell metastasis [53]. Nuclear translocation of STAT3 has been reported to be critical for the induction of the epithelial–mesenchymal transition (EMT) and increasing metastasis of bladder cancer, and this is mediated via SENP3 as a regulatory factor [54]. Circ-BGN and circ-RPPH1 are able to stimulate STAT3 signaling to promote gastric and lung tumor cell progression, respectively [55, 56]. Due to the important function of STAT3 in oncogenesis, studies have focused on the use of antitumor agents targeting this molecular signaling pathway to suppress it and impair tumorigenesis [57]. Fangchinoline increases oxidative stress to suppress STAT3 signaling to reduce myeloma progression [58]. Moreover, epigallocatechin-3-gallate decreases STAT3 expression by impairing colon tumor cell invasion and metastasis [59]. According to these descriptions, the function of STAT3 signaling in cancer is oncogenic and its suppression may therefore introduce new therapeutics for tumor therapy. The aim of the current review is to understand the function of STAT3 signaling in HCC, which will be discussed in detail in the next sections.

### A summary of STAT3 inhibitors

Repurposed drugs and natural products can be considered as important STAT3 inhibitors in cancer therapy [60]. In addition, medicinal chemistry has emerged as a new field for STAT3 suppression and cancer therapy [61]. The variety of natural products is large and they have shown high potential for modulating the STAT3 signaling pathway in cancer therapy. Betulinic acid, curcumin, plumbagin, diosgenin, caffeic acid, honokiol, and thymoquinone are among the phytochemicals that suppress STAT3 [62]. However, since natural products have poor bioavailability, the development of effective small molecule inhibitors of STAT3 has been proposed. These inhibitors are able to regulate upstream modulators of STAT3 such as JAK or Src, or they can directly interfere with the phosphorylation of STAT3 [63]. Most STAT3 inhibitors bind to the SH2 domain of STAT3 to interfere with its tyrosine phosphorylation [64]. Interestingly, different types of small molecules such as AG490, LS-104, INCB018424, and CEP-701 have been used in preclinical models and clinical trials [65]. However, since there are similarities between the SH2 domain of STAT3 and other members of the family, it is recommended that SH2 domain regulators be used with more caution in clinical trials.

## STAT3 in HCC apoptosis

One of the programmed cell death mechanisms important for cancer therapy is apoptosis. This cell death mechanism involves intrinsic and extrinsic pathways, with the intrinsic pathway involving mitochondria, while the extrinsic pathway involves death receptors [66]. In both pathways, the caspase cascade is upregulated to stimulate apoptosis. However, one of the drawbacks in cancer therapy is the development of apoptosis resistance, in which tumor cells do not respond to this form of cell death, leading to chemoresistance [67–70]. The interplay of oncogenic molecular signaling pathways and their upregulation may lead to the development of apoptosis resistance in HCC cells and inhibition of this intracellular mechanism. High expression of AKR1C3 suppresses apoptosis in HCC cells, and its silencing promotes apoptosis. To this end, AKR1C3 increases the expression of STAT3 via IL-6, and there is also a positive feedback loop in which overexpressed STAT3 promotes AKR1C3 expression in inhibiting apoptosis in HCC cells [71]. When apoptosis is inhibited, cancer cells gain more potential to proliferate and increase their population [72]. The upregulation of TRIM52 increases the growth rate of HCC cells, and to this end, TRIM52 stimulates STAT3 signaling as an oncogenic factor to prevent apoptosis in tumor cells [73]. Indeed, the function of STAT3 signaling is to protect HCC cells from apoptosis and to provide optimal conditions for tumor cell growth. However, stimulation of STAT3 signaling in HCC cells is complicated and requires interactions between different molecular signaling pathways. For example, circRNA-9119 is involved in protecting HCC cells from apoptosis. Upregulation of circRNA-9119 in HCC cells leads to inhibition of miR-26a to stimulate the JAK1/STAT3 axis to prevent apoptosis in tumor cells [74]. In chemotherapy, the main goal is to stimulate apoptosis to reduce HCC cell viability and progression. Doxorubicin (DOX) is commonly used in the treatment of HCC, and the goal of its administration is to induce apoptosis in tumor cells. The high expression level of CKLF1 can suppress apoptosis in DOX-exposed HCC cells, which is due to the activation of the IL-6/STAT3 axis [75].

Inhibition of apoptosis following upregulation of STAT3 may also lead to the development of radioresistance. Therefore, researchers have sought to understand apoptosis regulation after targeting STAT3 signaling in HCC therapy. XL888 is a selective inhibitor of HSP90 that can reduce the expression of STAT3 to stimulate apoptosis after inadequate radiotherapy in the treatment of HCC [76]. The factors targeting STAT3 signaling in HCC may affect tumorigenesis. miR-383 is an inducer of apoptosis in HCC cells. The expression of miR-383 decreases in HCC cells, while IL-17 shows an increase in expression. miR-383 downregulates the expression of IL-17, inhibiting STAT3 signaling in triggering apoptosis in HCC cells [77]. Another important factor regulating cancer cell progression is PDIA3, the upregulation of which leads to an unfavorable prognosis [78]. Inhibition of PDIA3 is important for suppressing growth and metastasis in multidrug-resistant tumor cells [79]. Low expression of PDIA3 leads to apoptosis in HCC cells, and after its inhibition, suppression of STAT3 phosphorylation occurs to stimulate apoptosis [80]. According to these studies, inhibition of apoptosis occurs frequently in HCC, and upregulation of STAT3 increases tumorigenesis and prevents apoptosis in tumor cells.

## STAT3 in HCC autophagy

In the previous section, the role of STAT3 in regulating apoptosis as a form of programmed cell death was explained. Another important mechanism is autophagy, which can have both oncogenic and oncosuppressive functions, and whose function is important in HCC. AMPK, Beclin-1, LC3, PI3K, and ATGs are important regulators of autophagy, which is a multistep mechanism involving initiation, elongation, maturation, and fusion steps. Therefore, targeting autophagy is of great importance in cancer therapy. One of the most important challenges is the dual function of autophagy as a pro-survival or pro-death mechanism [81–83]. Recent studies have shown that autophagy regulates the progression of HCC cells [84, 85]. Hepatocytic p62 impairs tumor progression and carcinogenesis via mTORC1 induction and defective autophagy [86]. Downregulation of SPTBN1 promotes the expression of YAP and inhibits autophagy in promoting HCC progression [87]. This section focuses on the role of STAT3 signaling in modulating autophagy in HCC. Capsaicin promotes the formation of reactive oxygen species (ROS), to increase STAT3 expression and induce autophagy. Notably, suppression of ROS /STAT3/autophagy enhances the ability of capsaicin to stimulate apoptosis in HCC cells [88]. Moreover, Zingiberensis newsaponin reduces the expression of AKR1C to suppress the Janus kinase 2 (JAK2)/STAT3 axis, thereby inhibiting autophagy and reducing the malignancy of HCC cells [89].

Under these circumstances, autophagy has an oncosuppressor function to inhibit cancer progression, and induction of autophagy is crucial to reduce HCC cell progression. Bufothionine decreases the serum level of IL-6 to inhibit the JAK2/STAT3 axis and increase the expression of ATG5, ATG7, and LC3II in autophagy induction and prevent the progression of HCC [90]. However, the function of autophagy in cancer can always be pro-survival, even after stimulation by agents and drugs. Myricetin increases MARCH1 levels to induce STAT3 signaling in mediating autophagy. Moreover, inhibition of autophagy increases the potential of myricetin to induce cell cycle arrest, demonstrating the function of autophagy as a mechanism promoting survival [91]. Dimethyl fumarate impairs HCC cell progression by suppressing growth, angiogenesis, and autophagy by increasing SOCS3 expression, thereby inhibiting the JAK1/STAT3 axis [92].

**Fig. 2:**
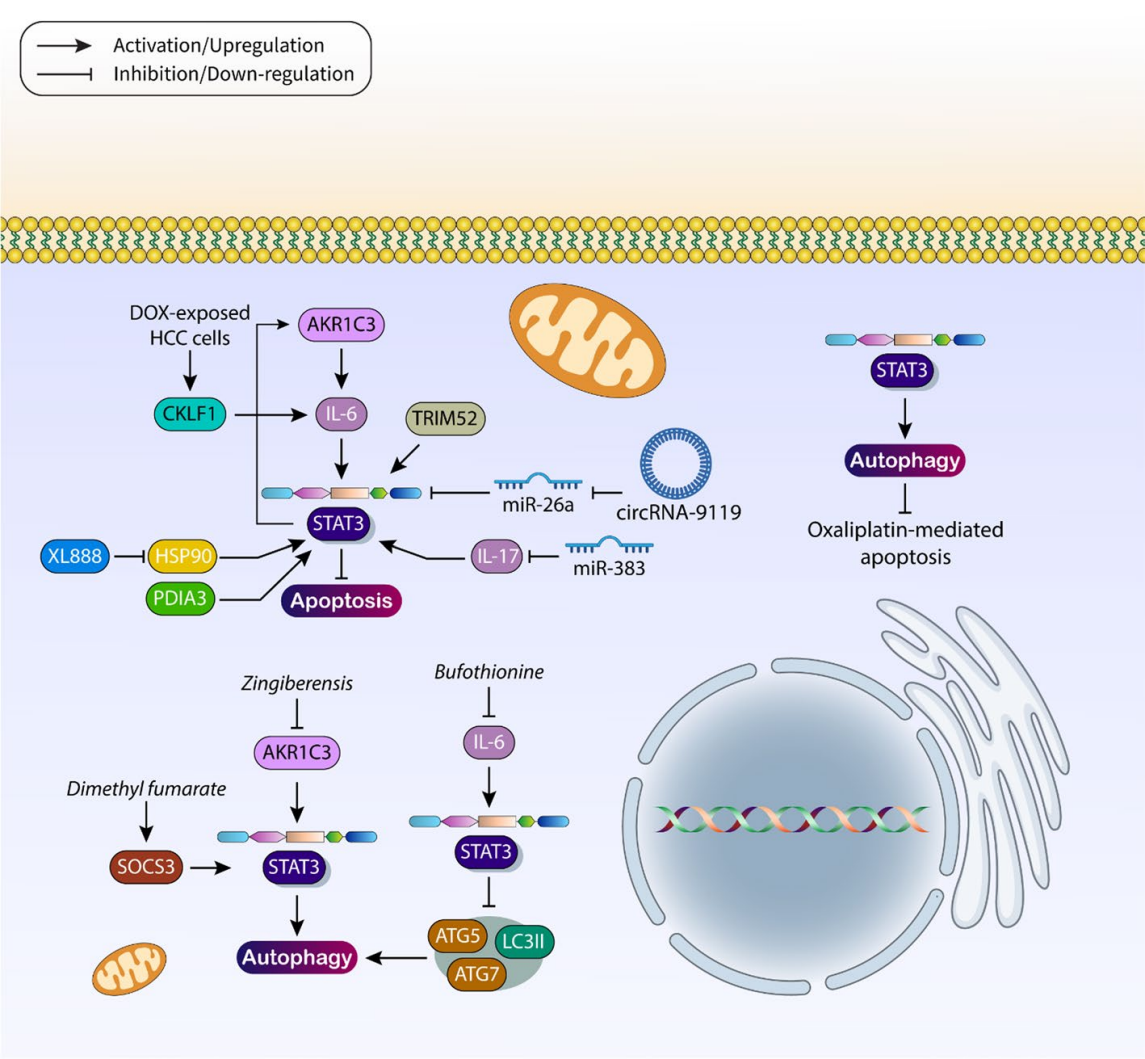
STAT3 signaling in the regulation of apoptosis and autophagy in HCC

Oxaliplatin is one of the chemotherapeutic agents commonly used in cancer treatment, although its efficacy may be determined and regulated by the autophagy mechanism. Stimulation of apoptosis and autophagy by 6-shogaol enhances the potential of oxaliplatin in cancer therapy [93]. Moreover, wogonin stimulates autophagy and increases the cytotoxicity of oxaliplatin [94]. However, induction of survival-promoting autophagy may lead to oxaliplatin resistance in HCC cells. Upregulation of STAT3 stimulates autophagy, whereas inhibition of the JAK2/STAT3 axis inhibits autophagy, promoting oxaliplatin-mediated apoptosis in HCC cells [95]. According to these studies, the interplay between STAT3 and autophagy not only determines the progression and survival rate of HCC cells, but also influences the response to chemotherapy. When autophagy has a tumor suppressive function, its induction is followed, and when it has a pro-survival function, its inhibition can promote apoptosis in HCC cells (Fig. 2 and Table 1).

**Table 1:** The role of STAT3 in regulating autophagy in HCC

Molecular pathway	Remark	Reference
ROS/STAT3/autophagy	Suppression of ROS /STAT3/autophagy promotes induction of apoptosis in HCC cells	[88]
AKR1C/JAK2/STAT3	Downregulation of AKR1C leads to inhibition of the JAK2/STAT3 axis to suppress autophagy in impairing tumorigenesis	[89]
JAK2/STAT3/autophagy	Bufothionine promotes ATGs and Beclin-1 in autophagy induction by inhibiting JAK2/STAT3 signaling to reduce tumorigenesis	[90]
MARCH1/STAT3/autophagy	Upregulation of MARCH1 stimulates STAT3 signaling to mediate autophagy	[91]
SOCS3/JAK1/STAT3	Dimethyl fumarate increases SOCS3 expression to inhibit the JAK1/STAT3 axis and suppress autophagy in HCC therapy	[92]
JAK2/STAT3/autophagy	Inhibition of JAK2/STAT3 suppresses autophagy and promotes oxaliplatin-mediated apoptosis in HCC cells	[95]

## STAT3/EMT axis in HCC

The previous sections have shown that STAT3 signaling is able to increase proliferation and survival of HCC cells via inhibition of apoptosis and modulation of autophagy (the function of autophagy can be pro-survival or pro-death). Although proliferation is an important hallmark of HCC cells, abnormal metastasis of these tumor cells may also adversely affect patient survival and prognosis. According to clinical and experimental reports, interfering with STAT3 signaling is a therapeutic approach to prevent HCC cell metastasis. On the other hand, the best-known mechanism for cancer invasion and metastasis is the epithelial–mesenchymal transition (EMT), which converts epithelial cells into mesenchymal cells and is associated with downregulation of E-cadherin and upregulation of N-cadherin and vimentin [96, 97]. The increase in metastasis and the development of chemoresistance may be due to the induction of EMT in tumor cells [98–100]. This section focuses on the function of STAT3 signaling in regulating the EMT mechanism in HCC. First of all, two important aspects should be considered regarding the role of STAT3 signaling in the regulation of HCC metastasis. In the initial phase, STAT3 signaling may be involved in the increased invasion and progression of HCC cells, either alone or by targeting related factors of EMT. For example, high expression of STAT3 leads to upregulation of transforming growth factor-beta (TGF-β1) to stimulate the EMT mechanism and enhance tumor metastasis [101]. In the next phase, the cooperation of STAT3 signaling with other molecular signaling pathways is required for EMT induction in HCC. DYRK1A may be involved in increasing metastasis of HCC cells via EMT induction. To this end, DYRK1A promotes the expression of STAT3 and accelerates its nuclear translocation; DYRK1A also interacts with TSC1 to phosphorylate the Smad2/Smad3 complex. Subsequently, it translocates to the nucleus, and the interaction of the Smad2/Smad3 complex with STAT3 signaling induces EMT and enhances metastasis of HCC cells [102]. Moreover, STAT3 can create positive feedback loops with upstream mediators that facilitate HCC cell metastasis and invasion. High levels of DDR1 lead to poor prognosis and low survival in HCC. DDR1 increases phosphorylation of STAT3, and overexpressed STAT3 in turn increases DDR1 expression. These interactions lead to EMT induction, which promotes HCC invasion [103]. Regarding the oncogenic function of STAT3 in increasing metastasis of HCC cells, the upstream factors that suppress STAT3 signaling may impair tumorigenesis. PIRK4 is considered an inhibitor of HCC invasion. In this way, PIRK4 impairs the phosphorylation of STAT3, suppressing EMT and metastasis in HCC [104].

PRN2 is a new emerging target in the field of cancer therapy because its downregulation impairs growth and metastasis and promotes apoptosis in cancer cells [105]. Moreover, PRN2 interacts with EGFR to promote cancer growth [106]. STAT3 is strongly regulated by PRN2, and its upregulation leads to radioresistance [107]. PRN2 promotes the expression of STAT3 in HCC and increases its nuclear translocation to induce EMT in HCC invasion and metastasis [108]. More importantly, endoplasmic reticulum (ER) stress may lead to malignancy of HCC cells via affecting STAT3 signaling. Hepatitis B virus small surface antigen leads to ER stress in HCC cells. Subsequently, ATF4 is upregulated to upregulate FGF19. The secreted FGF19 binds to the FGFR4 receptor on the cell surface to activate the JAK2/STAT3 pathway. Subsequently, nuclear translocation of STAT3 signaling occurs, increasing the levels of Slug, Snail, ZEB1, and Twist upon EMT induction and promoting tumor metastasis [109]. Due to advances in the field of biology, key molecular signaling pathways regulating STAT3 have become increasingly well understood, revealing both oncogenic and oncosuppressive properties. For example, EFTUD2 stimulates NF-κB to mediate inflammation and colitis-induced carcinogenesis [110, 111]. In HCC, high levels of EFTUD2 are indicative of poor tumor cell prognosis [112]. Mechanistically, EFTUD2 upregulates STAT3 expression, which is important for inducing EMT and facilitating metastasis and invasion of HCC [113].

**Fig. 3:**
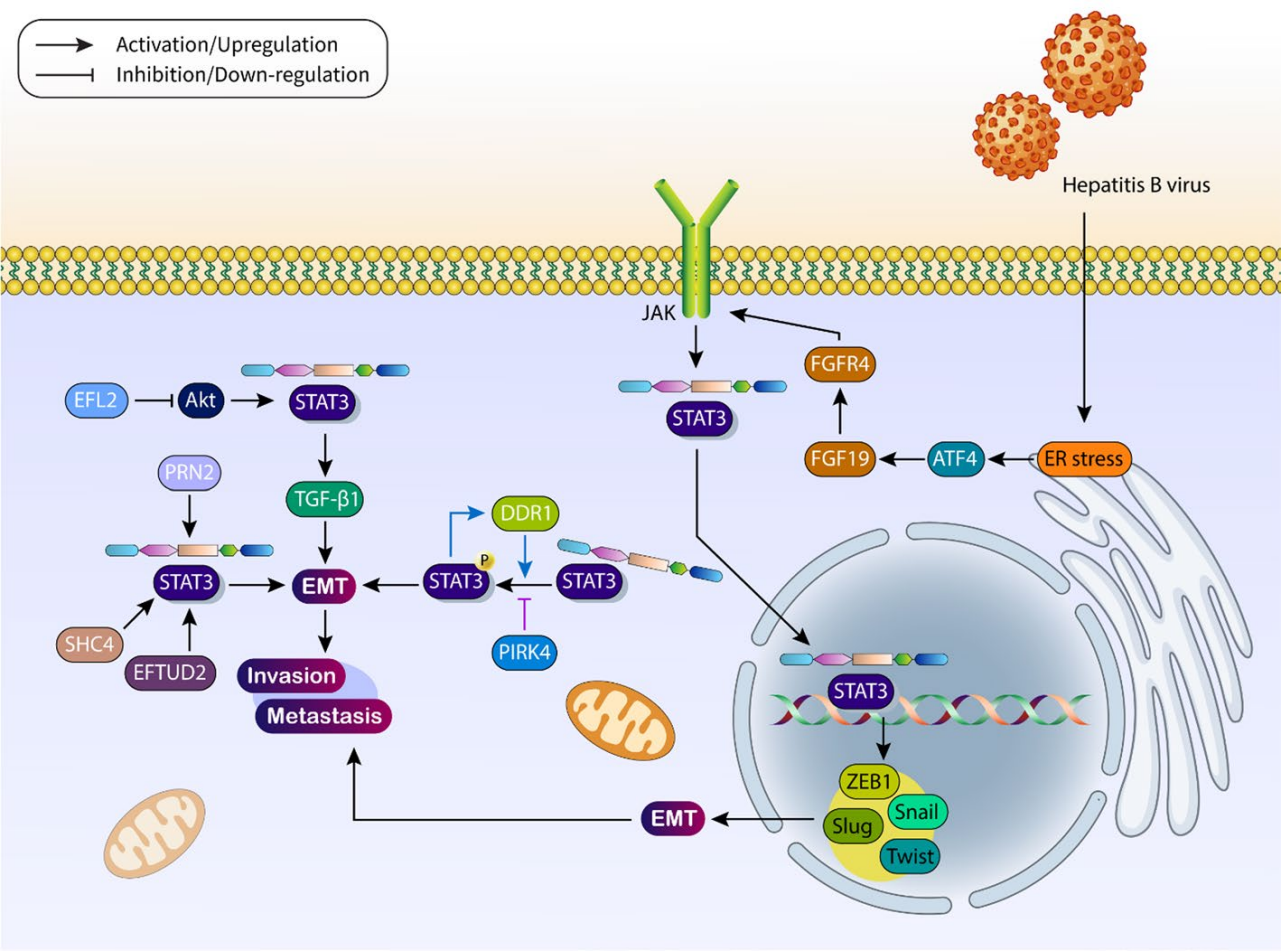
EMT mechanism regulation by STAT3 in HCC

Akt is another important factor in HCC, in which its overexpression and interaction with various molecular signaling pathways are responsible for the increase in HCC progression, malignancy, and development of drug resistance [114–116]. Interfering with Akt signaling is important for the treatment of HCC. Euphorbia factor L2 (EFL2) suppresses TGF-β-induced EMT in HCC cells. To this end, EFL2 reduces Akt expression and suppresses STAT3 signaling, which impairs tumor cell progression and metastasis [117]. Moreover, high expression of SHC4 has been associated with upregulation of STAT3 and subsequent induction of EMT in HCC cells [118]. According to these studies, STAT3 is a positive regulator of EMT in HCC, and therefore, suppression of STAT3 signaling may impair tumor progression by reducing EMT (Table 2 and Fig. 3) [119–122].

**Table 2:** The regulation of EMT mechanism by STAT3 in HCC cells

Signaling network	Remark	Reference
MiR-345/mTOR/STAT3/Akt	MiR-345 reduces the expression of IRF1 to suppress the mTOR/STAT3/Akt axis in inhibiting EMT	[222]
ERO1α/S1PR1/STAT3/VEGF-A	ERO1α increases the expression of S1PR1 to induce STAT3/VEGF axis in angiogenesis induction and EMT stimulation	[223]
DLGAP1-AS1/miR-26a/b-5p/IL-6/JAK2/STAT3	Downregulation of miR-26a/b-5p by DLGAP1-AS1 to induce STAT3 signaling and mediate EMT	[224]
STAT3/NFE2L1/STX12	Mitochondrial respiratory defect leads to STAT3 upregulation to induce NFE2L1/STX12 axis in EMT induction and facilitate tumor invasion	[225]
TLX3/STAT3/SNAI1/EMT	TLX3 reduces STAT3 expression to suppress SNAI1-mediated EMT	[226]
B7-H3/JAK2/STAT3/Slug	B7-H3 induces the JAK2/STAT3 axis to increase Slug expression upon EMT induction	[227]
KIAA1217/STAT3/EMT	KIAA1217 stimulates EMT mechanism via STAT3 upregulation to increase cancer progression	[228]
FEZF1-AS1/JAK2/STAT3	FEZF1-AS1 stimulates the JAK2/STAT3 axis during EMT induction	[229]
IL-35/STAT3/EMT	IL-35 promotes STAT3 expression to stimulate EMT	[230]
RBM3/STAT3/EMT	RBM3 promotes STAT3 expression to induce EMT	[231]
Glycochenodeoxycholic acid/STAT3/EMT	Up-regulation of STAT3 to induce EMT	[232]
STAT3/Snail/EMT	High expression level of Oct4 and Nanog promotes STAT3 expression to upregulate Snail in inducing EMT	[233]
STAT3/CASC11/PTEN/PI3K/Akt	STAT3 increases CASC11 expression to induce PI3K/Akt signaling via PTEN down-regulation upon EMT induction	[234]
IL-6/STAT3/HIF-1α/SNAI1/EMT	IL-6 promotes STAT3 expression to upregulate HIF-1α Up-regulation of SNAIL1 to induce EMT	[235]
DSCR8/miR-98-5p/STAT3/HIF-1α	DSCR8 promotes STAT3 expression via miR-98-5p sponging to increase HIF-1α expression and induce EMT, which enhances cancer invasion	[236]
STAT3/Twist/EMT	STAT3 increases the expression of Twist to stimulate EMT	[237]
TRIM27-USP7/STAT3/EMT	TRIM27-USP7 promotes STAT3 expression during EMT induction	[238]
STAT3/EMT	STAT3 stimulates EMT during increasing HCC invasion	[239]

## STAT3 in HCC drug resistance

The process of drug resistance in HCC is complicated and subject to the control of different molecular pathways in tumor cells. The process of drug resistance in HCC is favored by the upregulation of ribosomal RACK1, which increases tumor cell proliferation and viability [123]. PBK/TOPK expression increases in chemoresistant HCC cells and mediates oxaliplatin resistance via downregulation of PTEN expression [124]. In addition, high levels of CPEB1 decrease the stemness of HCC cells and are critical for suppressing drug resistance [125]. LINC01234 is able to promote the expression of MAGEA3 via miR-31-5p sponging to increase the proliferation rate of tumor cells and mediate drug resistance in HCC [126]. When the TRIM37 level is increased in HCC cells, it promotes Akt signaling to mediate chemoresistance [127]. Moreover, ID-1 is involved in triggering oxaliplatin resistance in HCC by inducing the pentose phosphate pathway [128]. Therefore, aberrant expression of proteins and genes can lead to the development of chemoresistance in HCC cells [129, 130], and this part of the text is focused on understanding the role of STAT3 signaling in the development of HCC drug resistance. The STAT3 pathway contributes to the development of drug resistance in HCC, and its expression level can be modulated by upstream mediators. The expression of DNMT3B is increased in HCC and shows a positive association with Oct4, which in turn increases the expression of IL-6 to induce STAT3 signaling in the development of sorafenib resistance in HCC and mediate an unfavorable prognosis in tumor cells [131]. As mentioned previously, the oncogenic function of STAT3 may be related to the inhibition of apoptosis in HCC cells. When dovitinib is administered, it suppresses STAT3 signaling in a SHP-1-dependent manner to prevent apoptosis and develop sorafenib resistance in HCC [132]. In the same way, dovitinib also increases the sensitivity of HCC cells to TRAIL and tigatuzumab. To this end, dovitinib inhibits STAT3 signaling in a SHP-1 manner to prevent tumor cell progression [133]. The function of STAT3 in the development of chemoresistance in HCC was confirmed by the finding of increased sensitivity of HCC cells to sorafenib after suppression of STAT3 signaling by pharmacological compounds or genetic tools [134]. Although studies have shown that activation of STAT3 signaling can lead to the development of sorafenib resistance in HCC, it has been shown that STAT3 expression is also regulated by sorafenib. Based on this finding, sorafenib administration downregulates STAT3 to prevent the development of TRAIL resistance in HCC [135]. In addition, the inhibition of STAT3 signaling by sorafenib is important for increasing the radiosensitivity of HCC cells [136].

High excretion of drugs can also lead to the development of chemoresistance, and drug efflux transporters may contribute to this condition [137]. ABCB1, also known as P-glycoprotein (P-gp), is a member of the ABC protein family and is located on the cell membrane [137, 138]. The substrates with a molecular weight of 250–1250 Da, including phospholipids, sterols, cholic acids, peptides, metabolites, and drugs, can be transported out of cells by ABCB1 [139]. In particular, STAT3 shows some interactions with ABCB1 in HCC cells. High expression of ABCB1 may lead to exocytosis of envatinib from HCC cells and thus development of drug resistance. Overexpression of EGFR in HCC cells induces STAT3 signaling to increase ABCB1 expression in the development of chemoresistance [140]. As mentioned earlier, the discovery of STAT3 is related to inflammatory processes. Now, the question arises whether inflammation in the liver can trigger STAT3 signaling and whether there is a link with the development of chemoresistance? The answer is yes. In a fibrotic liver, the presence of inflammation can lead to the induction of STAT3 signaling, which promotes the progression (proliferation and invasion) of HCC cells and mediates sorafenib resistance [141].

One of the important regulators of STAT3 signaling in HCC is RFX-1, which upregulates SHP-1 expression to suppress STAT3-mediated HCC progression [142]. SC-2001 is involved in disrupting HCC progression and promotes RFX-1 expression to upregulate SHP-1 in inhibiting STAT3 signaling and suppressing sorafenib resistance in tumor cells [143]. Even antitumor agents increase the level of SHP-1 in affecting the malignancy of HCC. Phloretin is a regulator of molecular signaling pathways in cancer [144] and can stimulate apoptosis to reduce tumor progression [145]. Phloretin suppresses the progression of HCC and promotes the expression of SHP-1 to suppress STAT3 signaling, leading to sorafenib sensitivity in tumor cells [146]. Moreover, inhibition of STAT3 signaling is important for the sensitivity of HCC cells to TRAIL-mediated apoptosis [147]. These studies suggest that high expression of STAT3 promotes drug resistance in HCC cells. Therefore, therapeutic targeting of this molecular pathway may impair tumorigenesis and promote chemosensitivity. In addition, regulators of STAT3 signaling may indirectly target STAT3 expression to modulate drug sensitivity in HCCs. Table 3 summarizes the role of STAT3 signaling in the development of drug resistance in HCC.

**Table 3:** The role of STAT3 signaling in developing drug resistance in HCC

Molecular pathway	Remark	Reference
STAT3/Mcl-1	Inhibition of the STAT3/Mcl-1 axis promotes tumor cell sensitivity to 5-fluorouracil	[240]
DANCR/IL-6/STAT3	DANCR promotes IL-6 levels to induce STAT3 signaling in the development of sorafenib resistance	[241]
Gankyrin/STAT3	Gankyrin stimulates STAT3 signaling in mediating sorafenib resistance in tumor cells	[242]
STAT3/PTTG1	Falcarindiol suppresses STAT3/PTTG1 axis in increasing cisplatin sensitivity of tumor cells	[243]
STAT3	Suppression of STAT3 signaling by YC-1 is important in enhancing drug sensitivity of tumor cells	[244]
STAT3	Inhibition of STAT3 signaling by NSC 74,859 is significant in enhancing the anticancer activity of cetuximab	[245]
MAEL/Akt/STAT3	MAEL stimulates the Akt/STAT3 axis to increase stemness and mediate sorafenib resistance	[246]
miR-589-5p/STAT3	miR-589-5p reduces the expression levels of SOCS2, SOCS5, PTPN1, and PTPN11 to induce STAT3 signaling in doxorubicin resistance	[247]
Let-7a/STAT3	Let-7a reduces STAT3 expression and increases the sensitivity of HCC cells to cetuximab	[248]
RhoE/ROCK2/IL-6/STAT3	Downregulation of RhoE leads to upregulation of ROCK2 to induce STAT3 signaling in developing chemoresistance	[249]
HOTAIR/STAT3/ABCB1	HOTAIR induces STAT3 signaling to increase ABCB1 expression in the development of cisplatin resistance	[250]
MAPK/ERK/STAT3	Metformin promotes the cytotoxicity of sorafenib by suppressing the MAPK/ERK/STAT3 axis	[251]

## STAT3 in HCC radio-resistance

**Fig. 4:**
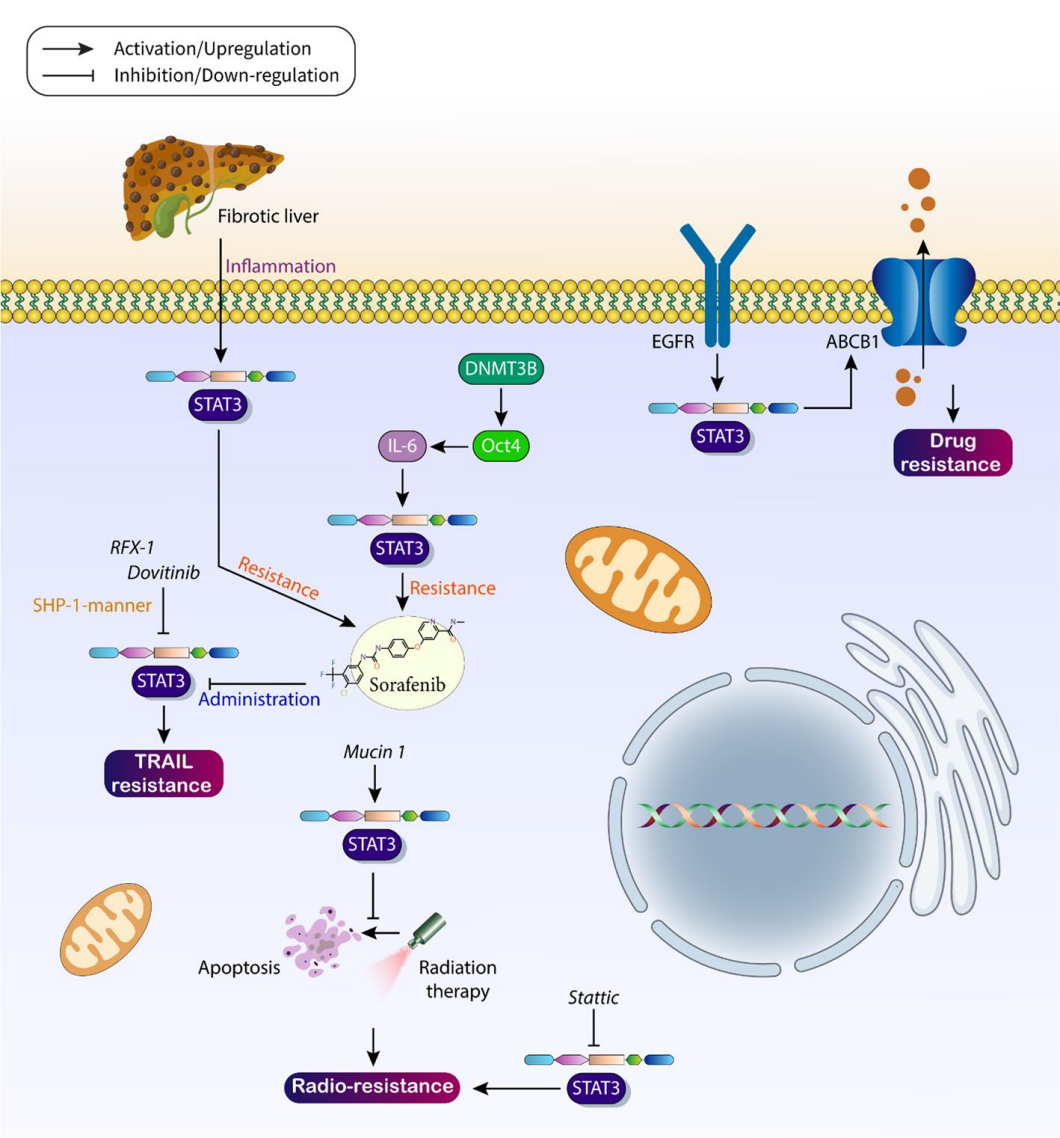
STAT3 in the development of chemoresistance and radioresistance in HCC

Radiotherapy is considered minimally invasive in the treatment of cancer, and is preferred to chemotherapy and surgery in some cases [148]. In addition, the development of stereotactic body irradiation and heavy ion therapy has greatly improved the potential of radiotherapy [148–150]. Although radiotherapy has brought many improvements in the treatment of patients with HCC, its potential may be threatened by the development of resistance. Molecular interactions have been reported to play an important role in the development of radiation resistance in HCC. Upregulation of NEAT1 may lead to radiation resistance in HCC due to stimulation of the PINK1/Parkin axis [151]. Furthermore, loss of CPS1 may lead to deubiquitination of c-Myc, triggering radioresistance in HCC [152]. Thus, when the expression level of oncosuppressor factors such as PTEN decreases and/or when oncogenic factors such as long noncoding RNA regulator of reprogramming (lncRNA ROR) increase, the likelihood of developing radioresistance in HCC is quite high [148, 153]. The role of STAT3 in the development of radioresistance in HCC has been investigated. High expression of mucin 1 may lead to radioresistance in HCC. Mechanistically, mucin 1 stimulates the JAK2/STAT3 axis to prevent apoptosis during radiation exposure in HCC cells [154]. Stattic, a small-molecule inhibitor of STAT3, is considered an inhibitor of radioresistance in HCC as it decreases STAT3 levels to suppress the radiation-mediated increase in metastasis of HCC cells [155]. However, the role of STAT3 signaling in regulating the response to radiotherapy in HCC needs further discussion (Fig. 4).

## Non-coding RNAs regulating STAT3 in HCC

### microRNAs

Noncoding RNAs (ncRNAs) play a rather crucial role in the process of tumorigenesis and microRNAs (miRNAs) are endogenous short noncoding RNAs that modulate gene expression by binding to the 3′-untranslated region (UTR) of target genes [156–158]. The role of miRNAs in HCC has been investigated and suggests a regulatory role of miRNAs in the progression and therapeutic response in HCC [159, 160]. Therefore, miRNAs are potential therapeutic targets in HCC. Moreover, miRNAs are able to regulate STAT3 signaling in various cancers [161, 162]. This section focuses on the role of miRNAs in STAT3 regulation in HCC. The progression of HCC cells can be suppressed by miR-637. Exogenous leukemia inhibitory factors (LIF) can induce STAT3 signaling and thus enhance HCC progression. On the other hand, miR-637 reduces the expression of LIF, to suppress STAT3 signaling [163]. miR-124 is another factor mainly responsible for reducing the progression of HCC cells. Of note, miR-124 downregulates STAT3 expression, and restoration of STAT3 expression impairs the efficacy of miR-124 in suppressing HCC cell proliferation and progression [164]. Two important references should be mentioned: First, upstream regulators of STAT3 can be modulated by miRNAs, and second, the goal of miRNAs in STAT3 targeting is to affect their downstream targets. For example, miR-340 reduces the expression of JAK1 to suppress STAT3 signaling and decreases the expression of Bcl-2, cyclin D1, and MMP-2 [165]. In addition, the level of miRNAs can be affected by STAT3 in HCC. Stimulation of STAT3 signaling leads to upregulation of Snail and Twist1 in facilitating metastasis of HCC cells. However, miR-370-3p binds to the 3′-UTR of Snail and Twist1, suppressing HCC metastasis. On the other hand, IL-8 stimulates STAT3 signaling to reduce miR-370-3p expression in mediating tumorigenesis [166]. Moreover, STAT3 increases the level of miR-23a to prevent gluconeogenesis in HCC [167]. miR-26a is a suppressor of HCC with attenuated expression in tumor cells [168]. miR-26a is able to reduce the expression of ERα to suppress the progression and proliferation of hepatomas mediated by E2 [169]. Moreover, miR-26a is involved in reducing DNMT3B expression in alleviating HCC progression [170]. Thus, miR-26a is an important tumor suppressor factor. miR-26a is able to decrease the levels of IL-6 by inhibiting STAT3 signaling and reducing the progression of HCC [171]. Apoptosis, proliferation, and invasion of HCC cells are tightly regulated by STAT3 signaling. High levels of STAT3 can lead to acceleration of proliferation through upregulation of c-Myc, an increase in migration through upregulation of MMP-9, and decreased apoptosis of cells through downregulation of Bax and caspase-3. However, miR-378a-3p is able to suppress STAT3 signaling and thus disrupt HCC progression [172]. Even the regulation of STAT3 by miRNAs can affect the response of HCC cells to therapy. miR-539 inhibits STAT3 signaling to stimulate apoptosis and promote the sensitivity of HCC cells to arsenic trioxide therapy [173]. Thus, STAT3 is strongly regulated by miRNAs in HCC cells.

### Long noncoding RNAs

Long noncoding RNAs (lncRNAs) are RNA transcripts that have gained much interest in recent years and regulate muscle differentiation [174], pluripotent stem cell reprogramming [175], apoptosis, and migration [176]. lncRNAs are responsible for aberrant expression of genes in tumors and may influence colony formation, metastasis, and malignancy of cancer cells [177–180]. Interestingly, lncRNAs have shown their potential in regulating STAT3 signaling in HCC. To this end, the lncRNA TPTEP1 reduces the phosphorylation of STAT3, which has a positive effect on HCC cell progression [181]. On the other hand, the lncRNA TINCR is able to enhance the progression of HCC. TINCR interacts and binds with TCPTP to stimulate STAT3 signaling, which is critical for enhancing tumor cell proliferation and metastasis [182]. Similar to miRNAs, the expression levels of which can be regulated by STAT3, STAT3 is able to bind to the promoter of lncRNAs to modulate their expression levels. STAT3 increases the level of lncRNA HOXD-AS1, which downregulates the level of miR-130a-3p by acting as competing endogenous (ce)RNA. Then, it induces SOX4 expression to upregulate EZH2 and MMP-2 to promote carcinogenesis in HCC [183]. In addition, infection with HBV can affect the expression level of lncRNAs in HCC. For example, in tissues infected with HBV, upregulation of lncRNA 01,152 is observed to increase the level of IL-23, inducing STAT3 signaling and promoting HCC progression [184].

The lncRNA 00,364 was reported to suppress the progression of HCC cells. Of note, lncRNA 00,364 suppresses STAT3 phosphorylation, paving the way for increased levels of IFIT2, leading to induction of apoptosis, cell cycle arrest in G1/S phase, and inhibition of proliferation [185]. However, most studies have focused on the function of oncogenic lncRNAs and their ability to induce STAT3 signaling. The lncRNA TUG1 is another factor whose overexpression has been observed in HCC and can relieve miR-144. This stimulates the JAK2/STAT3 axis, which promotes the growth and metastasis of HCC cells and the carcinogenesis process [186]. These studies have highlighted the fact that lncRNAs can modulate STAT3 signaling in HCC and their interaction is mainly based on influencing miRNAs [187]. It is proposed that small interfering (si)RNA, small hairpin (sh)RNA, and CRISPR/Cas9 can be used as powerful genetic tools to target lncRNAs in the treatment of HCC and suppress tumorigenesis.

### Circular RNAs

**Fig. 5:**
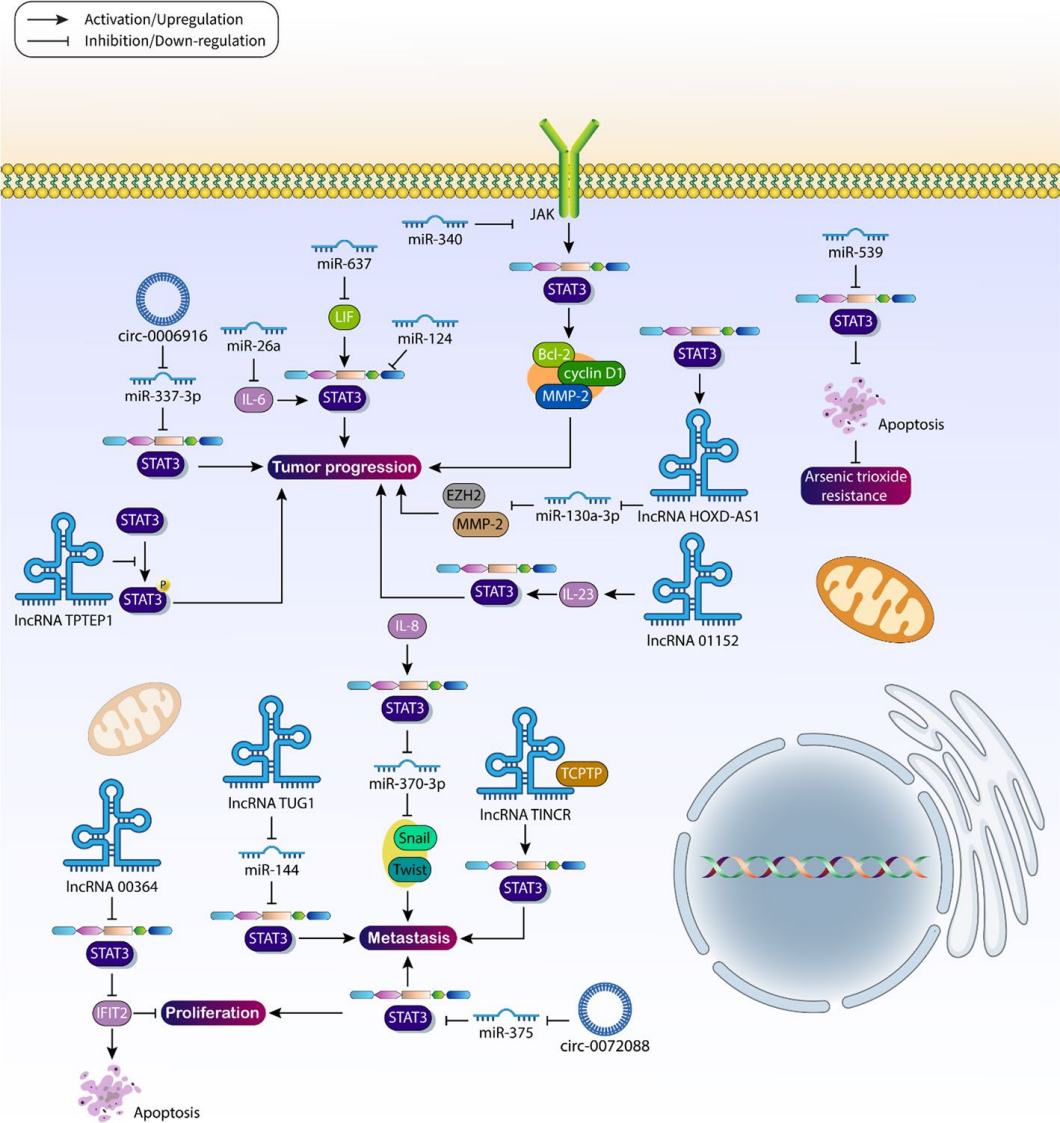
Regulation of STAT3 signaling by noncoding RNAs in HCC

Circular RNAs (circRNAs) are another member of the family of ncRNAs that do not code for proteins and develop a loop structure without 5′-3′ polarity, and with no polyadenylated tail in their structure [188, 189]. An abnormal amount of circRNAs is responsible for the process of tumorigenesis [190]. Moreover, circRNAs play a key role in HCC. For example, circ-0008934 sponges miR-1305 to increase TMTC3 expression, promoting HCC progression [191]. Moreover, circ-HIPK3 reduces the levels of miR-124 and miR-506 to increase PDK2 expression, thereby accelerating HCC progression [192]. Therefore, understanding the function of circRNAs is important for HCC therapy [193]. The malignancy of HCC cells is increased by the function of circ-0006916. It has been reported that circ-0006916 stimulates STAT3 signaling via downregulating miR-337-3p to increase the malignancy of HCC and mediate poor prognosis [194]. Circ-0072088 has an oncogenic function in cancers, and by decreasing miR-377 expression, circ-0072088 increases the progression of esophageal cancer cells [195]. Moreover, circ-0072088 increases NOVA2 expression via downregulating miR-377-5p, accelerating the progression of lung tumor cells [196]. In HCC, circ-0072088 shows a similar function and promotes tumorigenesis. A high level of circ-0072088 is associated with upregulation of STAT3, and this is achieved by downregulation of miR-375 to increase proliferation and metastasis of HCC cells and mediate EMT [197]. On the other hand, there are circRNAs that can inhibit the progression of HCC. Circ-0004913 is an inhibitor of HCC progression and for this purpose, it reduces the expression of miR-184 to increase HAMP expression. When the expression level of HAMP increases in response to circ-0004913, it can suppress proliferation, invasion, and glycolysis in HCC cells [198]. Table 4 and Fig. 5 provide an overview of the ncRNAs that regulate STAT3 signaling in HCC.

**Table 4:** The role of noncoding RNAs in the regulation of STAT3 signaling in HCC

Molecular pathway	Remark	Reference
MiR-486-5p/IGF-1R/STAT3	MiR-486-5p reduces IGF-1R expression to suppress STAT3 signaling	[252]
MiR-MTCO3P38/STAT3/PTTG1/MYC	Inhibition of STAT3 signaling and downstream targets by miR-MTCO3P38	[253]
MiR-363/S1PR1/STAT3	MiR-363 reduces S1PR1 expression to inhibit STAT3 signaling	[254]
Gα12/miR-122/c-Met/STAT3	Gα12 decreases miR-122 expression through HNF4α	[255]
LINC01133/miR-199a-5p/annexin A2	c-Met induction to stimulate STAT3 signaling	[256]
Circ-0006916/miR-337-3p/STAT3	LINC01133 promotes the expression of annexin A2 via miR-199a-5p to induce STAT3 signaling	[194]
LINC01433/miR-1301/STAT3	Circ-0006916 promotes STAT3 expression via miR-337-3p sponging in tumorigenesis	[257]
MiR-337-3p/JAK2/STAT3	LINC01433 promotes STAT3 expression via inhibition of miR-1301	[258]
MiR-137/EZH2/STAT3	MiR-337-3p suppresses the JAK2/STAT3 axis in affecting HCC progression	[259]
MiR-30e/JAK1/STAT3	MiR-137 reduces EZH2 expression and inhibits STAT3 signalingmiR-30e inhibits the JAK1/STAT3 axis in suppressing carcinogenesis	[260]
LINC01287/miR-298/STAT3	LINC01287 induces STAT3 via downregulation of miR-298 in EMT induction	[261]
SNHG16/miR-4500/STAT3	SNHG16 increases STAT3 expression via miR-4500 sponging in tumorigenesis	[262]
MiR-500a-3p/STAT3	MiR-500a-3p stimulates STAT3 signaling in cancer stemness enhancement	[263]
Circ-LRIG3/EZH2/STAT3	Circ-LRIG3 increases STAT3 expression in an EZH2-dependent manner to promote tumorigenesis	[264]
MiR-506/STAT3	MiR-506 suppresses STAT3 signaling to enhance natural killer cell cytotoxicity	[265]
MiR-146a	STAT3 promotes miR-146a expression in inhibiting anti-tumor immune response	[266]
NEAT1/miR-485/STAT3	NEAT1 sponges miR-485 to induce STAT3 signaling	[267]
MiR-451/IL-6R/STAT3	MiR-451 suppresses STAT3 signaling to inhibit angiogenesis via lower VEGF expression	[268]
MiR-515-5p/IL-6/JAK/STAT3	MiR-515-5p inhibits STAT3 signaling in reducing HCC progression	[269]
MiR-135a-5p/PTPRD/STAT3	MiR-135a-5p induces STAT3 signaling via downregulating PTPRD to promote tumorigenesis	[270]
Circ-0072088/miR-375/STAT3	Circ-0072088 increases STAT3 expression via miR-375 sponging to enhance cancer progression	[197]

## Pharmacological regulation of STAT3 in HCC

The use of novel antitumor agents for the treatment of HCC has received much attention recently. There are a number of reasons for this. The first is that conventional drugs and compounds such as chemotherapeutics are no longer very effective in treating cancer due to resistance. Therefore, when new types of therapeutics are introduced for HCC, the prognosis and survival of patients can be greatly improved. Due to the emergence of the field of precision medicine, studies have focused on targeting specific molecular pathways in cancer therapy. Because the STAT3 pathway is oncogenic and promotes progression of HCC cells, studies have focused on using antitumor agents that target the STAT3 pathway in cancer therapy. One of the new agents that has been extensively used to treat HCC in recent years is quercetin, which inhibits Akt signaling and suppresses HCC invasion [199]. In addition, quercetin shows a synergistic effect with oncolytic adenoviruses, which upregulate TRAIL in apoptosis induction [200]. JAK /STAT signaling can be regulated by quercetin in HCC therapy [201]. Quercetin suppresses the progression of HCC both in vitro and in vivo. Specifically, quercetin stimulates apoptosis and autophagy, and suppresses metastasis and proliferation of HCC cells. These anticancer activities of quercetin are mediated by inhibition of JAK2/STAT3 signaling [202]. Another antitumor agent currently used in cancer therapy is curcumin, which stimulates apoptosis and cell cycle arrest, impairs metastasis, and increases chemosensitivity [203–205]. In HCC, curcumin impairs tumor cell progression by affecting molecular signaling pathways, and its efficacy can be enhanced by nanoparticle delivery [206–208]. Trichloroethylene can induce EMT to promote HCC cell progression and metastasis. However, curcumin suppresses the IL-6R/STAT3 axis to inhibit EMT-mediated metastasis in HCC and reduce tumor cell malignancy [209]. In the context of the current review, STAT3 signaling promotes both growth and metastasis in HCC cells. Therefore, a novel therapeutic approach targeting STAT3 signaling in the treatment of HCC should affect two important hallmarks of HCC cells. For example, administration of (−)-oleocanthal may suppress STAT3 signaling to impair HCC metastasis and proliferation [210]. However, most studies have focused on these two hallmarks. Since chemoresistance is common in HCC [211–213], the development of novel therapies to inhibit STAT3 signaling should help reverse drug resistance in HCC.

**Fig. 6:**
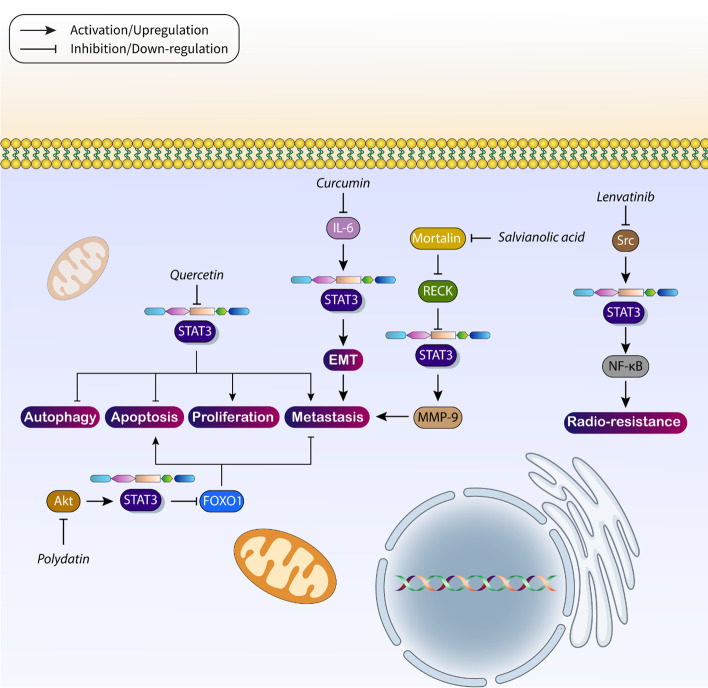
Antitumor compounds target STAT3 signaling in HCC

Polydatin, another reagent used in the treatment of HCC, has been shown to be efficient in apoptosis induction to suppress tumor cell proliferation and metastasis [214]. The antitumor activity of polydatin appears to be related to the inhibition of STAT3 signaling in HCC. Administration of polydatin reduces Akt expression to suppress STAT3 signaling as a downstream target. Subsequently, it is observed that overexpression of FOXO1 stimulates apoptosis and G2/8 M cycle arrest and reduces cancer cell metastasis [215]. In addition, regulation of STAT3 signaling by anticancer drugs is important to improve the response of HCC cells to radiotherapy. Since radioresistance is also common in HCC [216, 217], inhibition of radiosensitivity through STAT3 signaling can greatly enhance the therapeutic potential in HCC. Lenvatinib reduces the expression of Src to downregulate STAT3. It then inhibits NF-κB signaling to impair EMT and increase the radiosensitivity of HCC cells [218]. An important regulator of HCC progression is RECK, whose methylation by LINC01419 can increase tumor malignancy [219]. Moreover, GAS5 increases the expression of RECK in HCC suppression [220], indicating an anticancer effect of this factor. Salvianolic acid decreases mortalin levels to upregulate RECK. Subsequently, STAT3 signaling is inhibited to downregulate MMP-9 to delay HCC cell invasion and metastasis [221]. According to these studies, antitumor agents targeting STAT3 signaling may be very useful in the treatment and suppression of HCC (Table 5 and Fig. 6).

**Table 5:** The antitumor compounds targeting STAT3 signaling in HCC therapy

Compound	Molecular pathway	Remark	Reference
18-Glycyrrhetinic acid	STAT3/EMT	Inhibition of STAT3 signaling to suppress TGF-β-mediated EMT	[271]
LBH589	Gankyrin/STAT3/Akt	Reduction of proliferation and invasion by inhibition of the gankyrin/STAT3/Akt axis	[272]
Atorvastatin	IL-6/STAT3	Inhibition of the IL-6/STAT3 axis to induce senescence in tumor cells	[273]
Scutellarin	JAK2/STAT3	Inhibition of the JAK2/STAT3 axis to reduce cancer progression	[274]
Carnosic acid	STAT3 ERK1/2	Downregulation of STAT3 and ERK1/2 to suppress proliferation and invasion	[275]
Atiprimod	STAT3/NF-kB/apoptosis	Inhibition of the STAT3/NF-kB axis in the stimulation of apoptosis	[276]
Brusatol	STAT3/EMT	Inhibition of EMT by reducing STAT3 expression	[277]
Norcantharidin	JAK2/STAT3/TWIST	Inhibition of STAT3 signaling to reduce TWIST expression and suppress EMT	[278]
Sorafenib	TLR3/STAT3/SUMO1	Sorafenib reduces caspase-1 expression via suppression of the TLR3/STAT3/SUMO1 axis	[279]
ZnAS@SiO_2_ nanoparticles	SHP-1/JAK2/STAT3	Inhibition of STAT3 signaling to suppress EMT and reduce stemness	[280]
Hemistepsin a	STAT3	Inhibition of STAT3 to mediate apoptosis	[281]
Selenium sulfide	PLAGL2/C-MET/STAT3	Selenium sulfide inhibits the C-MET/STAT3 axis in a PLAGL2-dependent manner to induce apoptosis in tumor cells	[282]
Kahweol	Src/mTOR/STAT3	Kahweol inhibits the Src/mTOR/STAT3 axis in apoptosis induction	[283]
Sinomenine	AMPK/STAT3	Suppression of HCC progression through inhibition of the AMPK/STAT3 axis	[284]
Dihydrotanshinone	JAK2/STAT3	Inhibition of the JAK2/STAT3 axis in interfering with tumorigenesis	[285]
Isoliquiritigenin	ROS/MAPK/STAT3/NF-kB	Regulation of STAT3 signaling in a ROS-dependent manner to stimulate apoptosis in tumor cells	[286]
Liraglutide	IL-6/STAT3	Inhibition of STAT3 signaling to enhance antitumor immune response	[287]
Xanthin analog	ROS/JAK2/STAT3	Inhibition of STAT3 signaling in a ROS-dependent manner to stimulate apoptosis	[288]

## Conclusion and remarks

Thanks to advances in the field of cancer biology, the progression of cancer has been significantly affected. Nowadays, researchers increasingly understand the molecular mechanisms involved in the development of cancer cells. With a better knowledge of their interaction with other signaling networks, unique targeted therapies can be developed. One of the best known molecular signaling pathways involved in cancer development and progression is the STAT3 pathway. Although upregulation of STAT3 has been mentioned in several human cancers, its overexpression in HCC is unique because a number of biological behaviors are controlled by STAT3 signaling in HCC cells. HCC is the most common form of liver cancer, and its treatment is a major challenge for physicians around the world. STAT3 levels increase in HCC, and high STAT3 expression is associated with poor prognosis and malignant behavior of cancer cells. The biological aspect of STAT3 goes beyond a single molecular pathway, as STAT3 can interact with other signaling pathways such as EZH2. Nevertheless, ncRNAs are the most prominent modulators of STAT3 signaling in HCC. Upregulation of STAT3 is frequently observed during the progression of HCC and the interesting thing is that STAT3 can support tumor cells against apoptosis. The interaction of STAT3 with autophagy is complicated because autophagy has both oncogenic and oncosuppressive properties, and thus therapeutic interventions in autophagy should be undertaken with caution. The major mechanism promoting HCC invasion and metastasis is EMT, and it is noteworthy that EMT is activated by STAT3, leading to enhanced HCC progression. Moreover, high STAT3 levels may also lead to the development of radio- and chemoresistance in HCC. Therapeutic suppression of STAT3 signaling may impair progression and increase tumor cell sensitivity to therapy. Atorvastatin and brusatol are among the antitumor agents that target STAT3 signaling and can suppress the progression of HCC. In addition, ncRNAs create new molecular pathways in the regulation of STAT3. Future studies should focus on the clinical translation of experimental advances in ncRNAs.

The most important aspect of this review is to highlight both the underlying interactions of STAT3 with other molecular signaling pathways in HCC and the development of strategies to target it. The question now arises as to which part of the treatment of patients with HCC will be more important in the future. If there is a plan for developing effective therapeutics for HCC in the near future, it is better to focus on both parts. In fact, treatment strategies mainly use anticancer agents targeting STAT3 in HCC. Since combination therapy is a priority in HCC, it is proposed to use antitumor agents together with genetic tools targeting STAT3 and its downstream targets in HCC therapy.

One challenge physicians face in treating HCC in the clinical setting is that patients with HCC are diagnosed at an advanced stage. At this stage, tumor cells spread rapidly in the body and upregulation of STAT3 is one of the reasons for this. Therefore, STAT3 can be targeted by safe products in the treatment of HCC. One of the most important applications of STAT3 in patients with HCC is its function as a biomarker. Therefore, the expression of STAT3 may affect the prognosis of patients and also the response to therapy to prevent treatment failure.

In this comprehensive review article, the role of STAT3 in the progression of HCC has been discussed in detail. However, it is better to provide an overview of the function of STAT3 in HCC. First, STAT3 determines growth, metastasis, drug resistance, and radioresistance in HCC. Second, STAT3 interacts with upstream mediators in HCC, which include Akt, IL-6, PRN2, non-coding RNAs, TRIM52, and CKLF1. In addition, STAT3 can also regulate downstream signaling pathways, including TGF-β, ZEB1, Slug, and Twist, as well as matrix metalloproteinases (MMPs) and others. Interestingly, STAT3 can regulate important molecular mechanisms in HCC, including apoptosis, autophagy, and EMT, and can determine the response of HCC to chemotherapy and radiotherapy.

This paper has demonstrated that STAT3 has a versatile function in HCC due to its interaction with various networks and molecular signaling pathways. However, there are some limitations that should be considered for the future. The regulation of STAT3 in HCC has been clearly demonstrated. Moreover, its influence on downstream targets has been studied in great detail. However, one of the drawbacks of the current studies is that not enough attention has been paid to the role of STAT3 in the development of radioresistance. Moreover, one of the pathways for the transfer of STAT3 to HCC is its incorporation into exosomes, and this has been somewhat ignored in HCC. In addition, many clinical trials should be conducted worldwide in the future to investigate STAT3 serum levels and its association with prognosis and overall survival of patients. Another important aspect is that the anticancer drugs used to suppress STAT3 are mainly phytochemicals. Since STAT3 has binding sites, the discovery of drugs can be used to modulate STAT3 expression, and its suppression by small molecules can pave the way for the treatment of cancer patients.

## Data Availability

Not applicable.

## Abbreviations

3′-UTR: Three prime untranslated region
ABCB1: ATP-binding cassette subfamily B member 1
CircRNAs: Circular RNAs
EGF: Epidermal growth factor
EMT: Epithelial–mesenchymal transition
ER: Endoplasmic reticulum
HBV: Hepatitis B virus
HCC: Hepatocellular carcinoma
HCV: Hepatitis C virus
IL: Interleukin
JAK2: Janus kinase 2
LncRNAs: Long noncoding RNAs
MiRNAs: MicroRNAs
MMP: Matrix metalloproteinases
NcRNAs: Noncoding RNAs
SH2: Src homology 2
STAT3: Signal transducer and activator of transcription 3
TGF-β: Transforming growth factor-beta
